# *ARID1B* alterations identify aggressive tumors in neuroblastoma

**DOI:** 10.18632/oncotarget.17500

**Published:** 2017-04-28

**Authors:** Soo Hyun Lee, Jung-Sun Kim, Siyuan Zheng, Jason T. Huse, Joon Seol Bae, Ji Won Lee, Keon Hee Yoo, Hong Hoe Koo, Sungkyu Kyung, Woong-Yang Park, Ki W. Sung

**Affiliations:** ^1^ Samsung Genome Institute, Samsung Medical Center, Seoul, Republic of Korea; ^2^ Department of Translational Molecular Pathology, University of Texas MD Anderson Cancer Center, Houston, Texas, USA; ^3^ Department of Pathology, Samsung Medical Center, Sungkyunkwan University School of Medicine, Seoul, Republic of Korea; ^4^ Department of Genomic Medicine, University of Texas MD Anderson Cancer Center, Houston, Texas, USA; ^5^ Department of Pediatrics, Samsung Medical Center, Sungkyunkwan University School of Medicine, Seoul, Republic of Korea; ^6^ Department of Bioinformatics, Sungsil University, Seoul, Republic of Korea; ^7^ Department of Molecular Cell Biology, Sungkyunkwan University School of Medicine, Seoul, Republic of Korea

**Keywords:** ARID1B, ALK, MYCN, neuroblastoma, sequencing

## Abstract

Targeted panel sequencing was performed to determine molecular targets and biomarkers in 72 children with neuroblastoma. Frequent genetic alterations were detected in *ALK* (16.7%), *BRCA1* (13.9%), *ATM* (12.5%), *and PTCH1* (11.1%) in an 83-gene panel. Molecular targets for targeted therapy were identified in 16 of 72 patients (22.2%). Two-thirds of *ALK* mutations were known to increase sensitivity to ALK inhibitors. Sequence alterations in *ARID1B* were identified in 5 of 72 patients (6.9%). Four of five *ARID1B* alterations were detected in tumors of high-risk patients. Two of five patients with *ARID1B* alterations died of disease progression. Relapse-free survival was lower in patients with *ARID1B* alterations than in those without (*p* = 0.01). In analysis confined to high-risk patients, 3-year overall survival was lower in patients with an *ARID1B* alteration (33.3 ± 27.2%) or *MYCN* amplification (30.0 ± 23.9%) than in those with neither *ARID1B* alteration nor *MYCN* amplification (90.5 ± 6.4%, *p* = 0.05). These results provide possibilities for targeted therapy and a new biomarker identifying a subgroup of neuroblastoma patients with poor prognosis.

## INTRODUCTION

Neuroblastoma is the most common extracranial solid tumor in children. Half of all patients are classified as high-risk and demonstrate a poor prognosis. Although intensive multimodal treatment, including high-dose chemotherapy, improves clinical outcomes in patients with high-risk neuroblastoma, many patients still experience treatment failure [1] and have limited treatment options after relapse. Furthermore, high-risk neuroblastoma survivors suffer numerous long-term complications from previous intensive treatments [1, 2]. Novel treatment approaches are needed both to improve patient survival and to reduce treatment toxicities. Therefore, an emergent unmet clinical need in neuroblastoma is accurate prediction of outcome to facilitate the development of individualized treatment protocols.

Genomic analyses for pediatric neuroblastoma have identified recurrent somatic mutations in cancer-related genes such as *ALK, PTPN11, ATRX, MYCN*, and *NRAS* [3]. A recent analysis on structural variants using whole genome sequencing revealed that *TERT* promoter rearrangements characterize a subgroup of high-risk neuroblastoma with poor prognosis comparable to *MYCN* amplified tumors [4, 5]. *ARID1* alterations also reportedly predict poor outcome in patients with neuroblastoma [6].

In this study, we analyzed 72 cases of pediatric neuroblastoma with CancerSCAN™ (Supplementary Table 1) to find potential biomarkers to predict prognosis and identify patients likely to benefit from molecularly targeted therapies. CancerSCAN™ is a targeted deep sequencing panel and was developed mainly to identify genetic alterations for targeted therapy and the driver mutations of cancers.

## RESULTS

### Genomic profiling of neuroblastoma

**Figure 1:**
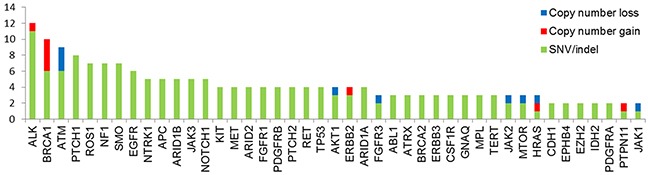
Mutation profiles of 72 patients with neuroblastoma Data are included for nonsynonymous single nucleotide variants as well as small insertion and deletion (SNVs/indels), and copy number (CN) gain and loss. Genes with more than one genetic alteration were included.

Tumor samples from 72 children with neuroblastoma were analyzed using targeted panel sequencing. At least one mutation in one of the 83 genes of the panel was found in 63 of 72 patients (87.5%). Across 83 genes in 72 tumor samples, we detected 180 single nucleotide variants (SNVs) and short insertions/deletions (indels) and 25 copy number variants (CNVs) (Supplementary Table 2). The prevalence of SNVs/indels and CNVs for each gene is shown in Figure 1. Alterations in *ALK* were detected in 12 of 72 patients (16.7%). Nonetheless, we did not detect any sign of *ALK* translocation. The second most common sequence alterations were in *BRCA1* (13.9%). Because *BRCA1* is located on chromosome 17q, copy number gain was also detected with other genes located in 17q in patients with 17q gain. In addition, six SNV/indels in *BRCA1* were detected with a range of allele frequency between 2.6∼34.0% (Supplementary Table 2) and predicted to be deleterious in function. The prevalence of somatic mutation in *BRCA1* remains to be elucidated in a larger study. Copy number loss in *ATM* was also associated with chromosome 11q deletion. In the *ATRX* gene, three novel missense mutations (A1988S, V2189A, and R498G) were detected. The mutation rate did not vary based on risk group (Supplementary Figure 1).

### Candidates for targeted therapies in neuroblastoma

Molecular target candidates for targeted therapy were detected in 16 of 72 patients (22.2%). Six cases with SNVs such as *ALK* R1275Q, F1174I, and R1192G, and one copy number gain in *ALK* could be potential candidates for *ALK* inhibitors [7–9]. Three R1275Q, an activating *ALK* mutation, were also confirmed with digital PCR method (Supplementary Figure 2) [10]. PARP inhibitors could be administered in 3 patients with *BRCA1* truncating mutations and 3 patients with *ATM* copy number loss [11–13]. In addition, *HRAS* Q61R, *MET* exon14 skipping mutation, *ERBB2* copy number gain, and *STK11* copy number loss were each detected in one patient, respectively (Supplementary Table 3).

### *ARID1B* and neuroblastoma

**Figure 2:**
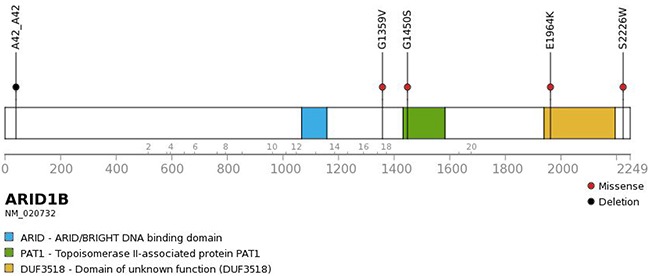
Genomic alterations affecting *ARID1B* Four missense mutations and one deletion were found in *ARID1B*.

**Figure 3:**
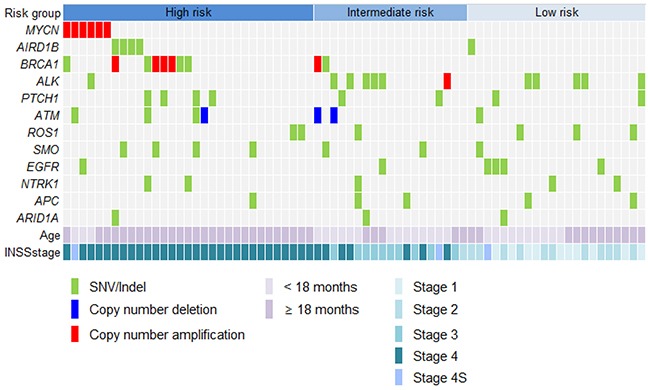
Targeted sequencing to identify genetic alterations Four of five patients with *ARID1B* mutations were classified as high-risk. *MYCN* amplification was measured by fluorescence in-situ hybridization because *MYCN* was not included in the panel.

**Figure 4:**
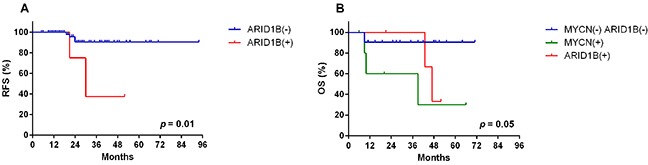
Relapse free survival and overall survival **(A)** Three-year relapse-free survival (RFS) in patients with and without *ARID1B* mutations was 37.5 ± 28.6% and 90.5 ± 4.5%, respectively (*p* = 0.01) (n = 72). **(B)** In high-risk patients, the 3-year overall survival in patients with *ARID1B* mutations, with *MYCN* amplification, or with neither *ARID1B* mutation nor *MYCN* amplification were 33.3 ± 27.2%, 30.0 ± 23.9%, and 90.5 ± 6.4%, respectively (*p* = 0.05).

Five patients showed sequence alterations in *ARID1B*, consisting of four missense mutations and one small deletion (Figure 2). Sanger sequencing confirmed all four missense mutations but the small deletion. Three of the four missense mutations were predicted to be deleterious and the remaining one to be neutral. Protein expression was maintained in tissues with *ARID1B* mutations. Four of five patients with the *ARID1B* mutation belonged to the high-risk group (Figure 3). Four SNVs were detected in *ARID1A* in three patients. In the present study, there was no patient who have both sequence alterations in *ARID1* and *MYCN* amplification (Figure 3). Only *ARID1B* gene mutation was associated with differential relapse-free survival (RFS) between patients with mutation and wild-type gene among genes listed in Figure 1. RFS at 3 years in patients with *ARID1B* mutations was lower than in those without (Figure 4A, *p* = 0.01). In the analysis of only high-risk patients, 3-year RFS in patients with (n = 4) and without *ARID1B* mutations (n = 27) was 37.5 ± 28.6% and 76.7 ± 10.2%, respectively (*p* = 0.25). Survival of patients whose tumors harbored *ARID1B* mutations, which was similar to that of patients with *MYCN*-amplified tumors, was significantly worse than that of high-risk patients with neither *MYCN* amplification nor *ARID1B* mutations (Figure 4B, *p* = 0.05). Median follow-up duration was 37 months in patients with *ARID1B* mutations, 20 months in patients with *MYCN* amplification, and 29 months in patients with neither *MYCN* amplification nor *ARID1B* mutations, respectively. Whereas three high-risk patients with relapsed tumors with neither *MYCN* amplification nor *ARID1B* mutations were rescued with salvage treatment after relapse, two relapsed patients with *ARID1B* mutations died of tumor progression.

## DISCUSSION

Although our approach is limited in its ability to detect alterations in non-coding regions or in genes outside our panel, the current panel covers genetic markers frequently involved in cancers and is sufficient to identify clinically relevant alterations in the context of targeted therapy in patients with neuroblastoma. *ALK* gene mutations were most frequent, as described in other studies [3, 14]. In addition, two thirds of *ALK* mutations were known to increase sensitivity to ALK inhibitors in the present study. In addition, we found druggable mutations in several other genes, including *BRCA1*, *ATM*, *HRAS*, *MET*, *ERBB2*, and *STK11*. Several targeted agents, including ALK inhibitors, are in clinical trials for treatment of neuroblastoma [15]. Recent studies for pediatric cancers demonstrate the promise of genome-guided therapy in children and adolescents although some challenges remain to be resolved such as rapid progression of disease and reemergence of resistance [16–18].

While mutation rates are higher in high-risk patients based on whole-genome sequencing, the mutation rate detected by panel sequencing was not different between risk groups [4]. The limited number of genes in the panel partly explains the results, however, involvement of major biologic pathways may also not vary between risk groups.

*ARID1* genes are integral components of the SWI/SNF neural progenitor-specific chromatin-remodeling BAF complex essential for the self-renewal and differentiation of multipotent neural stem cells [19]. The chromatin remodeling function of the SWI/SNF complex is crucial for the gene expression program that converts precursor cells to their terminally differentiated counter parts [20]. AT-rich interacting domain (ARID) and domain of Unknown Function 3518 (DUF3518) in ARID1B bind to DNA and interact with the helicase subunits in the BAF complex, respectively [21–24]. Accordingly, missense mutations in these domains are likely to disrupt DNA-bind ability and interaction between ARID1B, BRG1, and BRM, which presumably compromise the function of BAF complexes and diminish their ability to regulate gene expression [25, 26]. Overall, the complex has been suggested to be an important tumor suppressor in human cancer. Tumor-specific deletions encompassing *ARID1B* have been reported in central nervous system tumors [27], and multiple members of this complex have been identified as tumor suppressor genes in ovarian cancer, pancreatic cancer, liver cancer, and neuroblastoma [6, 19, 28–30]. According to Sausen et al. [6], disrupted BAF complex signaling may preserve an undifferentiated progenitor state, leading them to suggest that alterations in *ARID1* genes correlate with a more aggressive neuroblastoma phenotype. In the present study, four of five *ARID1B* mutations were found in high-risk patients, and two of them died due to progressive disease. Most studies about ARID1B-mediated disorders reported nonsense or frameshift mutations rather than missense mutation. However, this results were primarily from Coffin-Siris syndrome (CSS) cohorts and therefore might reflect ascertainment bias [26]. Most nonsense and frameshift mutations activate nonsense-mediated mRNA decay (NMD), therefore, these mutations are likely to cause NMD of the ARID1B transcript rather than the expression of the mutant ARID1B protein [31]. Truncating mutations that avoid NMD usually cause a distinct and more severe phenotype than that observed in NMD [32]. This finding suggests that there may be a different mutation pattern between CSS and cancers. TCGA data show that missense mutation is most frequent type of mutation in *ARID1B* in cancers. Although hemizygous deletion in *ARID1B* was the main type of mutation in neuroblastoma found in the previous study [6], all *ARID1B* changes were SNVs and small deletions in the present study. In recent study addressing the genomic landscape of schwannoma, 23% (23/99) of schwannoma harbored *ARID1B* mutations, in which only one mutation was frameshift deletion and others are missense mutations or non-frameshift indels [33]. The authors also proved maintenance of ARID1B expression in tissues with *ARID1B* mutation, which is probably due to remaining wild-type allele, therefore, suggest a haploinsufficient role of *ARID1B* in cancer. The effect of sequence alterations in the study need to be further validated. In addition, *ARID1* mutations were not found in patients with *MYCN* amplification, which is different from the results of a previous study [6] in which samples harboring both *MYCN* amplification and *ARID1* alteration were all of cell line origin. The further examination in a large population requires to validate the role of *ARID1B* mutation on prognosis and possible mutual exclusivity between *MYCN* and *ARID1* in neuroblastoma patients.

One limitation of this study is the lack of normal pair samples. The sequence alterations detected in this study do not exclude the possibility of low prevalence individual SNPs. We attempted to exclude the possibility by eliminating all sequence alterations known to be DNA polymorphisms in databases (dbSNP, Clinvar, SNPeffect 4.0, ESP5400, 1000 Genomes, Exac03, Korean SNP DB, and EVS) and publications. However, validation is necessary to further confirm the functions of each variant.

In conclusion, our results add to the current body of knowledge regarding the genomic characteristics of neuroblastoma. Around 20% of children with neuroblastoma might benefit from targeted therapy. In addition, we identified a subgroup of neuroblastoma with *ARID1B* mutation shows an aggressive behavior. These findings may provide a new biomarker to identify another subgroup of neuroblastoma with high-risk features. A neuroblastoma-specific panel using NGS could be developed to identify more comprehensive genomic information to molecularly treat and predict prognosis with sophistication based on genomic characteristics such as *MYCN, TERT, ATRX, ALK, ARID1*, and telomere length. In the near future, the risk stratification of patients with neuroblastoma can be changed based on genetic characteristics.

## MATERIALS AND METHODS

### Patients

Neuroblastoma tumor samples from 72 children diagnosed between 2008 and 2015 were included in this analysis (Supplementary Table 4). The proportion of formalin-fixed paraffin embedded (FFPE) tissue and fresh frozen tissue were 48.6% and 51.4%, respectively. Only specimens with > 30% tumor were included and the purity of tumor was more than 60% in 70% of samples. The study was approved by the Institutional Review Board of Samsung Medical Center (2014-08-060). All participants provided written informed consents.

### Deep sequencing using CancerSCAN™

Genomics DNA (250 ng) from each tissue was sheared in a Covaris S220 ultrasonicator (Covaris, Woburn MA, USA) and used for the construction of a library with CancerSCAN™ probes and a SureSelect XT reagent kit, HSQ (Agilent Technologies) according to the manufacturer's protocol. This panel was designed to enrich exons of 83 genes [34] covering 366.2 kb of the human genome. After enriched exome libraries were multiplexed, the libraries were sequenced using the 100-bp paired-end mode of the TruSeq Rapid PE Cluster Kit and TruSeq Rapid SBS kit on the Illumina HiSeq 2500 sequencing platform (Illumina Inc., San Diego, CA, USA). The DNA sequence data were aligned to the human genome reference (hg19) using the MEM algorithm in BWA 0.7.5 [35]. Duplicate read removal was performed using Picard v.193 and SAMTOOLS v0.1.18 [36]. Local alignment was optimized using the Genome Analysis Toolkit (GATK) v3.1-1 [37]. We also used BaseRecalibrator from GATK for base recalibration based on known single nucleotide polymorphisms (SNPs) and indels from Mills, dbSNP138, and 1000G gold standard, 1000G phase1 and Omni 2.5. Sequencing coverage is shown in Supplementary Table 5.

### Bioinformatic analysis

Variant calling was done only in regions targeted in CancerSCAN™ v1. We detected SNVs using three tools: MuTect 1.1.4, LoFreq 0.6.1, and SNVer 0.5.3 [38–40]. We filtered out falsely detected variants from abnormally aligned strand biased and clustered reads by in-house developed scripts. ANNOVAR was used for annotating the detected variants by diverse resources including dbSNP138, COSMIC, TCGA, EPS5400, 1000 Genomes, Exac03, and the in-house Korean SNP DB. Indels were detected by Pindel 0.2.4 [41] and annotated by ANNOVAR. To filter out germline variants, we applied two algorithms: i) Except for hotspot mutations, variants with an allele frequency greater than or equal to 97% were filtered out; ii) Suspect germline variants were filtered out based on whether the allele frequency was ≥ 1% of previously mentioned database or ≥ 3% of 480 samples from healthy Korean subjects.

We used CancerSCAN™ software (unpublished) to detect CNVs. In CancerSCAN™, the software ‘Depth of Coverage’ in GATK v3.1-1 was used to calculate sequencing coverage in each exon. The median of mean coverage for total exons was calculated and divided by the gained average for normalization of mean coverage of exons. The median value of normalized exons of pattern matched normal reference datasets (HapMap cell lines, FFPE tissues from normal patients) was used as a reference value, and then the normalized median value of exons of each patient was divided by the reference value and transformed to the binary logarithm, respectively. Tumor purity for adjusting CNVs was calculated using normalized coverage and B allele frequencies at all exons. Using normal samples, coverage of sample was fitted and after that major ploidies and copy number in the neutral region were decided. Based on the inferred copy neutral region and major ploidies, tumor purity was calculated using the relationship between B allele frequencies and tumor ploidy data. Finally, we defined ‘Copy number loss’ as when the copy number was less than one and ‘Copy number gain’ as when the copy number was more than four using the above method.

### *MYCN* amplification

Paraffin sections of each tumor were hybridized for interphase FISH studies, using commercial probes: Vysis LSI MYCN (2p24)/CEP 2 (2p11.1-q11.1) (Abbott Molecular, Abbott Park, IL, USA). DAPI was used to counterstain nuclei. The stained sections were examined by a microscope equipped with fluorescence filters (BX51, Olympus, Tokyo, Japan). The fluorescent signals of *MYCN* (green) and *CEP2* (orange) were counted in fifty non-overlapping nuclei of the tumor cells. *MYCN* amplification was defined as a 10-fold increase of the *MYCN* signal number compared to the reference probe located on chromosome centromere 2.

### Statistical analysis

For two group comparisons, Student's *t*-test was used to determine differences between mean values for normal distribution. Survival analysis was performed using the Kaplan-Meier method and survival differences between groups were examined by log-rank test. All data were analyzed for significance using GraphPadPrism 6 software (San Diego, CA, USA).

### URLs

dbSNP, www.ncbi.nim.nih.gov/SNP/; EVS, http://evs.gs.washington.edu/EVS/; ClinVar, www.ncbi.nih.gov/clinvar/; SNPeffct 4.0, http://snpeffect.switchlab.org/

## SUPPLEMENTARY MATERIALS FIGURES AND TABLES




