# Supplementary material for: ARID1B alterations identify aggressive tumors in neuroblastoma
Source: Oncotarget. 2017 Apr 28;8(28):45943–50. doi: 10.18632/oncotarget.17500 (PMC5542239; doi:10.18632/oncotarget.17500)
Supplement: Supplementary file 1 [file oncotarget-08-45943-s001.pdf]

ARID1B alterations identify aggressive tumors in neuroblastoma

SUPPLEMENTRY MATERIALS

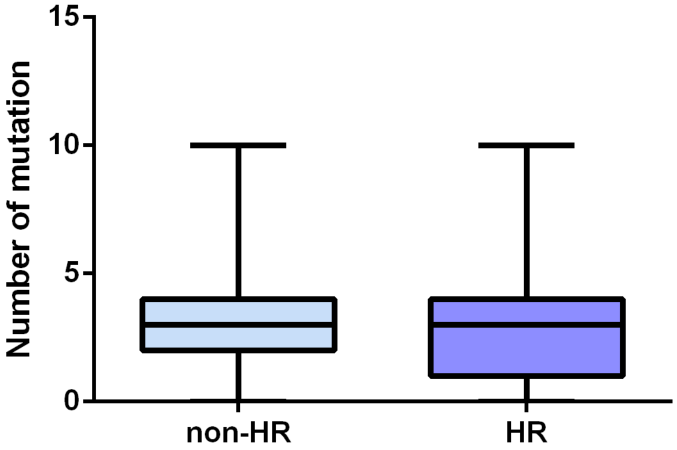

Supplementary Figure 1: The mutation rate was not different according to the risk group.

ID	Gene	Exon	AA change	dbSNP	Chr	Start	Ref	Alt	RefC	AltC	VAF (CancerSCAN)	VAF (dPCR)
NBL-44	ALK	exon25	R1275Q	rs113994087	chr2	29432664	C	T	323	31	**0.088**	**0.082**
NBL-39	ALK	exon25	R1275Q	rs113994087	chr2	29432664	C	T	746	50	**0.063**	**0.075**
NBL-27	ALK	exon25	R1275Q	rs113994087	chr2	29432664	C	T	599	74	**0.110**	**0.119**

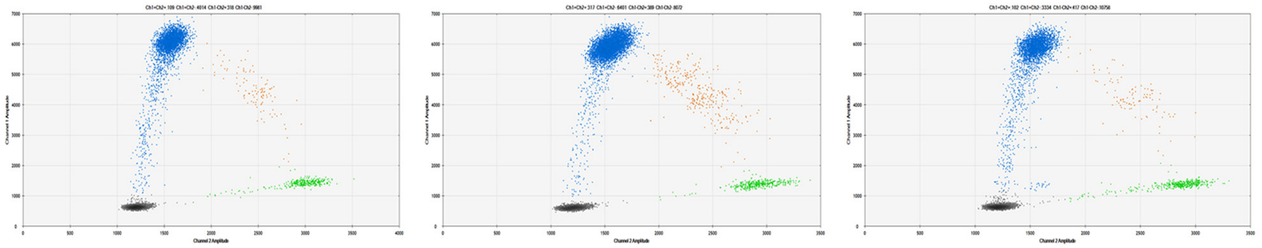

Supplementary Figure 2: Three R1275Q, an activating *ALK* mutation, were also confirmed with digital PCR method.

Supplementary Table 1: CancerSCAN™ v1.1.

See Supplementary File 1

Supplementary Table 2: SNV/Indel.

See Supplementary File 1

Supplementary Table 3: Genetic markers for targeted therapies.

See Supplementary File 1

Supplementary Table 4: Patient characteristics.

See Supplementary File 1

Supplementary Table 5: Sequencing coverage.

See Supplementary File 1
